# Selective and precise solid phase extraction based on a magnetic cellulose gold nanocomposite followed by ICP-OES analysis for monitoring of total sulfur content in liquid fuels†

**DOI:** 10.1039/d4ra04427d

**Published:** 2024-09-02

**Authors:** Kgomotso G. Mabena, Philiswa Nosizo Nomngongo, Nomvano Mketo

**Affiliations:** a Department of Chemistry, College of Science, Engineering and Technology (CSET), University of South Africa Florida Science Campus 1709 Johannesburg South Africa mketon@unisa.ac.za nomvano.mketo@gmail.com +27-114712032; b Department of Chemical Sciences, University of Johannesburg Doornfontein Campus, P.O. Box 17011 Johannesburg 2028 South Africa

## Abstract

This study describes the synthesis and characterization of a magnetic cellulose gold nanocomposite (MCNC@Au) for magnetic solid phase (m-SPE) extraction of total sulfur content in liquid fuel samples followed by analysis using inductively coupled plasma-optical emission spectroscopy (ICP-OES). The nanocomposite was prepared using an *in situ* co-precipitation method and characterization results from FTIR, P-XRD, TEM and SEM-EDX techniques confirmed the formation of the targeted nanocomposite. To achieve good extraction efficiency, the 2-level half-fractional factorial design and central composite design were used to investigate the most influential parameters of the proposed m-SPE method. The multivariate optimization results showed that efficient extraction was obtained when 27.5 mg sorbent mass, 35 minutes sorption time, 200 μL eluent volume and 8 min elution time were used. The optimal parameters resulted in excellent accuracy (98.8%), precision (1.7%), LOD (0.039 mg L^−1^), LOQ (0.129 mg L^−1^), MDL (0.014 μg g^−1^) and MQL (0.047 μg g^−1^). The optimized and validated m-SPE method was applied in real fuel oil samples, revealing a total sulfur content range of 13.20 ± 0.05–15.70 ± 0.02 μg g^−1^ for crude oil, 7.32 ± 0.01–9.12 ± 0.03 for μg g^−1^, 8.41 ± 0.02–9.15 ± 0.06 μg g^−1^ for gasoline and 9.10 ± 0.02 and 9.70 ± 0.04 μg g^−1^ for kerosene samples, sugesting high concentration levels of sulfur in crude oils. However, the obtained sulfur content levels are within the accepted standards in fuel oils, except for those of crude oil and kerosene samples. Therefore, the proposed m-SPE method followed by ICP-OES analysis has proven to be an alternative procedure for rapid and selective quantification of total sulfur in fuel samples.

## Abstract 2

This study describes the synthesis and characterization of a magnetic cellulose gold nanocomposite (MCNC@Au) for selective extracting total sulfur content in liquid fuel samples.

## Graphical abstract

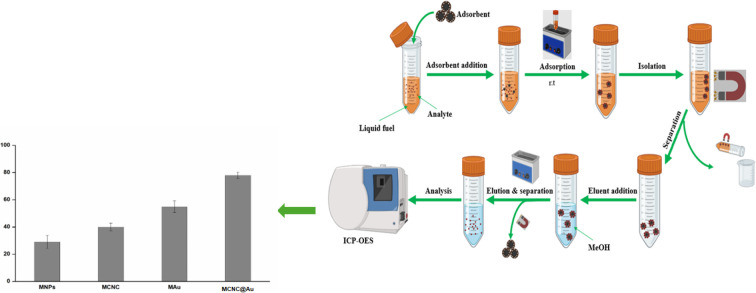

## 1. Introduction

Sulfur is a naturally occurring element in crude oil, hence it also appears in crude oil derivatives like gasoline, diesel, and kerosene, just to name a few.^1–4^ These fuel oils account for over 82% of the world's primary energy, with half produced from petroleum, and their daily consumption means sulfur poses a significant environmental threat because it contributes to the formation of sulfuric acid rain.^5–7^ To protect the environment, government departments worldwide have mandated ultra-low sulfur content levels (≤10 ppm) in fuels to refineries before commercialization to minimize sulfur-based air pollution.^8^ Refinery industries face a problem of their own since sulfur compounds in crude oil deactivate catalyst materials and cause corrosion in pipelines during the refining process.^8–10^ Therefore, from both an environmental and economic standpoint, it is crucial to develop rapid and effective analytical methods for monitoring sulfur concentration levels in fuel oils.

This project aims to use adsorptive desulfurization (ADS) as a potential method to determine the total sulfur content in liquid fuels. Using a solid sorbent, sulfur compounds are extracted to form the basis of ADS. Various adsorbents, such as activated carbons (AC),^11–13^ activated clay materials,^14^ mesoporous silica,^15^ metal–organic frameworks (MOFs),^6,16^ alumina, zeolites,^17,18^ and ion exchange resins,^19^ have been used for desulfurization, but they have low selectivity towards sulfur due to competitive adsorption with other organic compounds and require time-consuming isolation methods.^20^ Therefore, further research is needed to develop more suitable and less costly adsorbents for total sulfur determination in fuel oils.

Sample preparation is a crucial step in analytical chemistry as it enhances the analytical technique's sensitivity and selectivity before moving on to quantitative analysis. ICP-OES analysis is challenging for determining sulfur concentration levels in liquid fuel matrices due to potential issues like plasma thermal properties degradation when organic samples are introduced. Hence, extracting sulfur compounds in liquid fuels prior to the ICP-OES analysis is a crucial step of sample preparation.^20^ Liquid–liquid extraction (LLE) and solid-phase extraction (SPE) are two popular sample preparation methods that are used for preconcentration before analyses. Because LLE uses more toxic solvents and has lower preconcentration factors,^21,22^ SPE is recommended for preconcentration of a wide range of different analytes. SPE does have certain drawbacks, though, like the requirement to modify the SPE cartridges and the expensive and wasteful use of hazardous organic solvents during the elution and washing processes.^22,23^ The magnetic solid-phase extraction (m-SPE) technique is an improvement of a typical SPE technique, and it is employed in this study. The m-SPE is a technique for extracting and separating a target analyte from a solution that employs a magnetic adsorbent. The analyte is adsorbed into the magnetic sorbent and then easily separated using an external magnet and the recovered adsorbent is then used to elute the analyte. Most magnetic adsorbents are easily recyclable and reusable, which can save a significant amount of money and help the environment.^24,25^ These results show that m-SPE has numerous benefits, including simplicity, quickness, and environmental friendliness.

Herein, magnetic cellulose gold nanocomposite (MCNC@Au) was developed for the extraction of total sulfur content in liquid fuel samples prior to ICP-OES analysis. The presence of cellulose enhances the multifunctional adsorption capabilities, cost-effectiveness, and biodegradability of the developed magnetic adsorbent.^26,27^ Gold nanoparticles (AuNPs) have been investigated as promising materials for the adsorption of sulfur compounds due to their selectivity towards sulfur-containing compounds, taking advantage of their high chemical affinity towards sulfur atoms.^28,29^ Moreover, AuNPs are non-toxic and inert materials with unique chemical and physical properties and are easy to synthesize.^30–33^ To the best of our knowledge, AuNPs have not been previously studied as potential adsorbents for the preconcentration of sulfur in liquid fuels. However, AuNPs have been employed as potential adsorbents for the preconcentration of various organic compounds in various matrices. For example, Wang *et al.* reported the extraction and preconcentration of polyaromatic hydrocarbons (PAHs) in drinking water using AuNPs as adsorbent and this sample preparation method was named solid phase nanoextraction. The analytes were analyzed using either HPLC^34^ or laser-exciting^35^ time-resolved Shpol'skii spectroscopy and their method was successful, producing excellent analytical figures of merit, high recovery and low LODs. They further used the method to successfully recover monohydroxy-PAHs in urine samples with good LODs.^36^ The AuNPs were also reported by Shen and co-workers for preconcentration of alkanethiols and dithiols in saline solution prior to CE-LIF analysis and it was discovered that these nanoparticles had a high selective and extraction power for aminothiols.

Therefore, MCNC@Au nanocomposite was employed in this project as a potential adsorbent for preconcentration and extraction of total sulfur in selected liquid fuel samples. The most influential parameters of the proposed m-SPE method were investigated using a multivariate experimental design. Furthermore, the optimized and validated m-SPE method was applied to real commercial fuel oil samples (crude oil, gasoline, diesel and kerosene).

## 2. Experimental

### 2.1. Materials

Dry maize stalk waste (MSW) was provided by a local maize farm. The MSW was cut into small fragments, washed with ultrapure water, and dried in an air-circulating oven at 100 °C for 18 hours. The MSW fragments were then ground into a fine powder, sieved under a 30 mesh stainless strainer, and stored at room temperature in an airtight container. Methanol, sodium chlorite, acetic acid, potassium hydroxide, sulfuric acid and snakeskin dialysis tubes were of analytical grade. Ferric chloride hexahydrate (FeCl_3_·6H_2_O) ACS reagent, ferrous chloride tetrahydrate (FeCl_2_·4H_2_O), sodium citrate dihydrate ≥ 99% FG [HOC(COONa)(CH_2_COONa)_2_·2H_2_O], tetrachloroauric acid (HAuCl_4_·3H_2_O) ∼ 49% Au, sodium hydroxide (NaOH), 28 w/v% ammonium solution and methanol (99%) were purchased from Sigma-Aldrich (Johannesburg, South Africa). Crude oil CRM containing 15 822 ppm of sulfur was also purchased from Sigma-Aldrich (Johannesburg, South Africa). Ultrapure water which was obtained from a Milli-Q system was used throughout the experiments.

The real crude oil samples were obtained from one of the South African petrochemical companies. The commercial diesel and gasoline samples were purchased from two different local gas stations, whereas the commercial kerosene samples were purchased from a local supermarket. All the samples were stored in the refrigerator prior to analysis and were used as purchased. Prior to the total sulfur determination experiments, the samples were allowed to warm up to room temperature and were diluted using DCM.

### 2.2. Extraction of CNCs

The extraction of CNCs was done following modified reported methods.^27,37,38^ The MSW powder was firstly alkali treated with a KOH aqueous solution of 2% (w/w) for 4 h at 100 °C under mechanical stirring and then washed several times with distilled water until the alkali was completely removed, and finally dried at 60 °C for 24 h in an air-circulating oven. After this treatment, the fibres were bleached with a solution made up of equal parts (v/v) of acetate buffer (27 g NaOH and 75 mL glacial acetic acid, diluted to 1 L of distilled water) and aqueous chlorite (1.7 wt% NaClO_2_ in water). This bleaching treatment was performed at 80 °C for 6 h. The bleached fibres were washed repeatedly in distilled water until the pH of the fibres became neutral and subsequently dried at 40 °C for 24 h in an air-circulating oven. The fibre content throughout these chemical treatments was about 4–6% (w/w). A white powder was obtained which was labelled as treated maize stalk (TMS). The TMS was milled with a blender, passed through a 30 mesh screen and then used for the extraction of cellulose nanocrystals (CNCs) by acid hydrolysis. The hydrolysis was performed at 45 °C for 45 min under vigorous and constant stirring. For each gram of TMS, we used 15 mL of H_2_SO_4_ 9.17 M. Immediately following the hydrolysis, the suspension was diluted 10-fold with cold water to stop the hydrolysis reaction and centrifuged for 10 min at 8000 rpm to remove the acid excess. The snakeskin dialysis tube was then used to dialyze the obtained precipitate with tap water to remove non-reactive sulfate groups, salts, and soluble sugars until the neutral pH (∼4 days) was reached. Subsequently, the resulting suspension of the dialysis process was ultrasonicated for 10 min. The water was then removed through slow evaporation. The resulting precipitate was then dried overnight in an air-circulating oven to obtain shiny white cellulose nanocrystals (CNCs). The crystals were then dispersed into 250 mL of water in an airtight container to form CNC suspension and stored in a fridge for further experiments.

### 2.3. Synthesis of MCNC (Fe_3_O_4_@CNC)

*In situ* chemical co-precipitation method was used for the synthesis of magnetic cellulose nanocrystals (MCNC).^30,39–41^ This was done by adding 100 mL of CNC suspension into a 2-neck round bottom flask containing a magnetic stirrer. The suspension was then degassed for 15 minutes to remove oxygen gas. FeCl_3_·6H_2_O (5.2 g) and FeCl_2_·4H_2_O (2 g) were added to the suspension and allowed to stir for 1 hour at room temperature under inert (N_2_) conditions. The Fe^2+^/Fe^3+^ ratio was important for the control of morphology and the size of the nanoparticles to be formed. Then NH_4_OH (25%) was added dropwise to the mixture until a dark brown precipitate was formed. The reaction was allowed to stir for a further 1 hour. The precipitate was then separated from the supernatant by an external magnet and then washed four times using ultrapure H_2_O and ethanol. The composite was dried for 24 hours at 50 °C in an air-circulating oven.

### 2.4. Synthesis of MCNC@Au nanocomposite

The prepared MCNC were coated with gold (Au) shell by first adding 20 mL of HAuCl_4_·3H_2_O (50 mM) into a round bottom flask containing 100 mL of deionized water followed by boiling. 5 mL (1 mg mL^−1^) of the prepared MCNC suspension was added to the boiling gold solution. Sodium citrate (5 mL, 80 mmol L^−1^) was then added slowly into the solution under constant stirring. The colour of the solution changed from brown to dark red-purplish colour. The boiling continued for 5 minutes. The heat source was removed and stirring continued until the solution cooled to room temperature. The MCNC@Au nanocomposite was washed four times with ultrapure water and then separated using an external magnet. The nanocomposite was dried for 24 hours at 50 °C in an air-circulating oven.

### 2.5. Instrumentation

The functional groups of the nanoparticles and their structural changes were investigated using the Fourier-transformed infrared (FTIR) spectra with a Bruker Tensor 27 FTIR spectrophotometer. The synthesized samples were mixed with potassium bromide (KBr) before being compressed into pellets. During the analysis, data was collected from 400 to 4000 cm^−1^ region. The crystal structure of the nanoparticles was determined using powder X-ray diffraction (p-XRD). The XRD measurements were taken with a PANalytical X'Pert Pro powder diffractometer. The measurements were taken with Cu K radiation in the 2*θ* range from 5 to 90° at 40 kV and 40 mA operational conditions, the wavelength was 0.15405 nm. Predictions for the raw p-XRD patterns were made using OriginPro 8.5 software and the miller indexes were obtained using High Score (Plus) software and ICDD PDF-4+ 2015. The internal structure of the nanomaterials' properties such as morphology and particle size were studied using transmission electron microscopy (TEM). It was carried out using a Jeol JEM-2100F Field Emission Electron Microscope at an acceleration voltage of 200 kV (JEOL Inc., Akishima, Japan) equipped with a LaB_6_ power source. The TEM samples were dispersed in methanol and a drop of the mixture was transferred onto a TEM grid (Cu-grid, 200 mesh) coated with a lacy carbon film. The average particle size on the synthesized nanocomposite was calculated using a histogram obtained using OriginPro 8.5. A Tescan Vega 3LMH scanning electron microscopy (SEM) and the samples were carbon-coated using an Agar Turbo Carbon Coater. SEM was employed for imaging, morphology and elemental mapping of the nanomaterials. The extracted sulfur content was analysed using an Agilent Technologies 700 Series ICP-OES instrument containing an axially oriented torch and an Agilent Technologies SPS 3 autosampler for sample uptake. Table S1† lists the instrument's operational parameters and the wavelengths that were compatible with the sulfur element.

### 2.6. General procedure of the m-SPE method

**Fig. 1:**
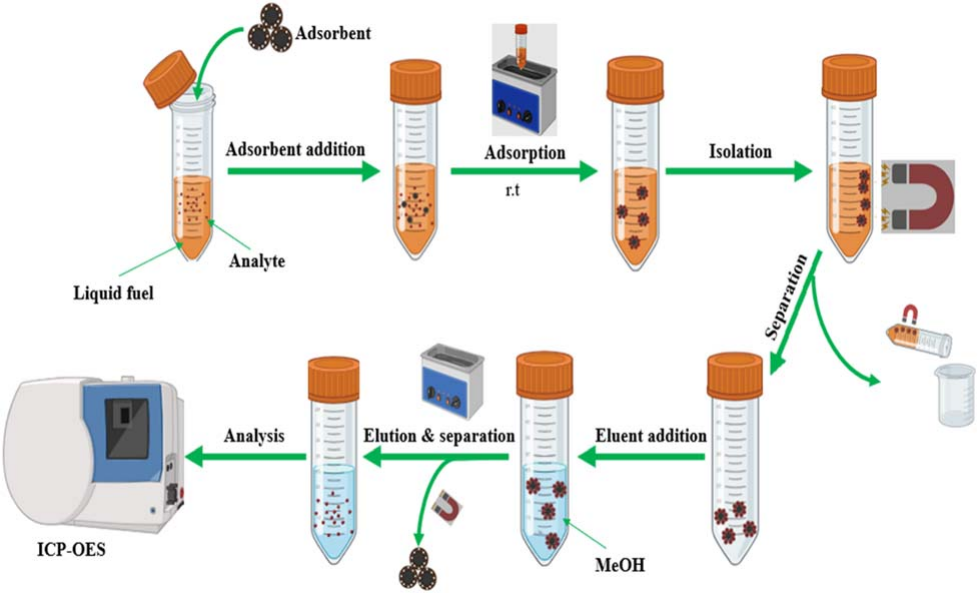
Graphical diagram for m-SPE method for extraction of sulfur in liquid fuel samples.

First, dichloromethane (DCM) was used to dilute crude oil CRM, which had 15 822 ppm of sulfur, down to 20 ppm. A 50 mL conical tube was filled with 20 mL of diluted crude oil, and 27.5 mg of the magnetic sorbent was then added. To carry out the m-SPE procedure (pretreatment), the mixture was sonicated for 35 minutes at room temperature. Following two DCM washes, the sulfur-impregnated sorbent was separated from the matrix using an external magnet (a neodymium–iron–boron alloy magnet). To perform the elution experiment, 200 μL of MeOH was added to a tube holding the sorbent that had been impregnated with sulfur. The tube was then left on the sonicator for eight minutes at room temperature. The magnetic sorbent was then separated from the eluent solution using an external magnet (see Fig. 1). Before the ICP-OES analysis, the eluent solution was transferred to a 15 mL conical tube and diluted to the 10 mL mark with deionized water. Following the ICP-OES analysis, the sulfur concentrations found in the diluted eluent solution were multiplied by a dilution factor of 50.

The extraction efficiency was used as a response and was calculated using the following equation (eqn (1)) :1
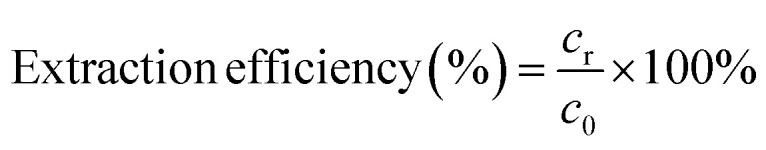
where *c*_r_ is the total sulfur concentration after elution (sulfur content removed) and *c*_0_ is the initial concentration of total sulfur in the sample before adsorption.

### 2.7. Multivariate optimization method

The screening process of the most significant factors was conducted using a 2-level half fractional design with five independent variables. The mass of the sorbent, sorption time, pH, eluent volume and elution time were chosen as the parameters to be varied. The minimum and maximum amount of each factor was chosen based on previous studies. Table S2† shows all the factors with the maximum and minimum amount for each factor. The response surface methodology was employed to further optimize the significant figures. The central composite design (CCD) serves as the foundation for the second-order response surface methodology. The insignificant parameters were maintained constant while the significant parameters from the first-order design were further optimized. Analytical results were interpreted using 3D surface plots from MiniTab software. The relationship between the parameters and the response is formulated on these plots. Additionally, each parameter's impact on the extraction efficiency was monitored.

## 3. Results and discussion

### 3.1. Characterization MCNC@Au and its components

#### 3.1.1. FTIR

**Fig. 2:**
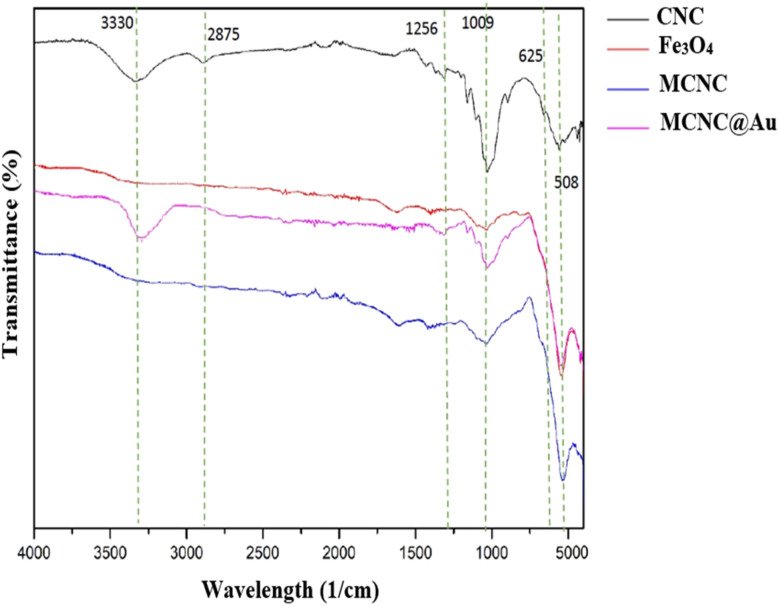
FTIR spectrums of CNC, Fe_3_O_4_, MCNC and MCNC@Au.

FTIR spectrum was used to confirm the identity of functionality attached to the nanoparticles. The spectrums are compared to confirm the functionality. Fig. 2 shows the four spectra of CNC, Fe_3_O_4_ (magnetite), MCNC and MCNC@Au. The spectra pattern of MCNC@Au (pink) was similar to the combined spectra of both pure CNC (black), Fe_3_O_4_ (red) and MCNC (blue) with slight changes on certain wavelengths. Some important characteristics functional groups of CNC were observed at 3330 cm^−1^ (–OH hydrogen bonding), 2875 cm^−1^ and 1256 cm^−1^ (stretching and deformation of CH_2_ bonding), 1009 cm^−1^ (C–O bonding) and 625 cm^−1^ (C–OH bonding). The peak at 508 cm^−1^ in *c* corresponded to the Fe–O bond stretching of Fe_3_O_4_. The signal at 625 cm^−1^ assigned to the C–OH of CNC was absent in FTIR spectra of MCNC@Au, indicating the reaction between the Fe ions and the hydroxyl groups of CNCs.

#### 3.1.2. TEM

**Fig. 3:**
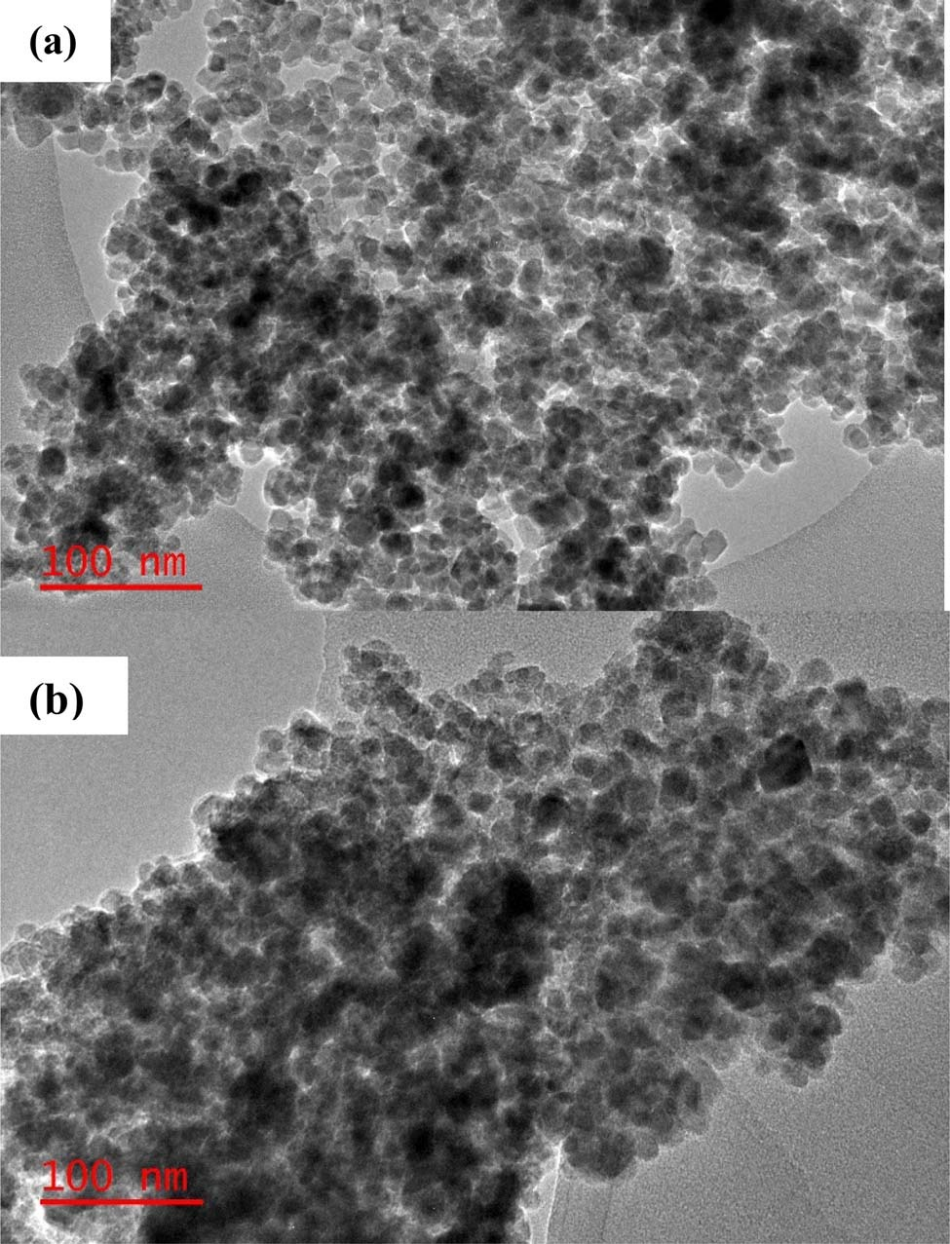
TEM Images of (a) MCNC and (b) MCNC@Au nanocomposite.

**Fig. 4:**
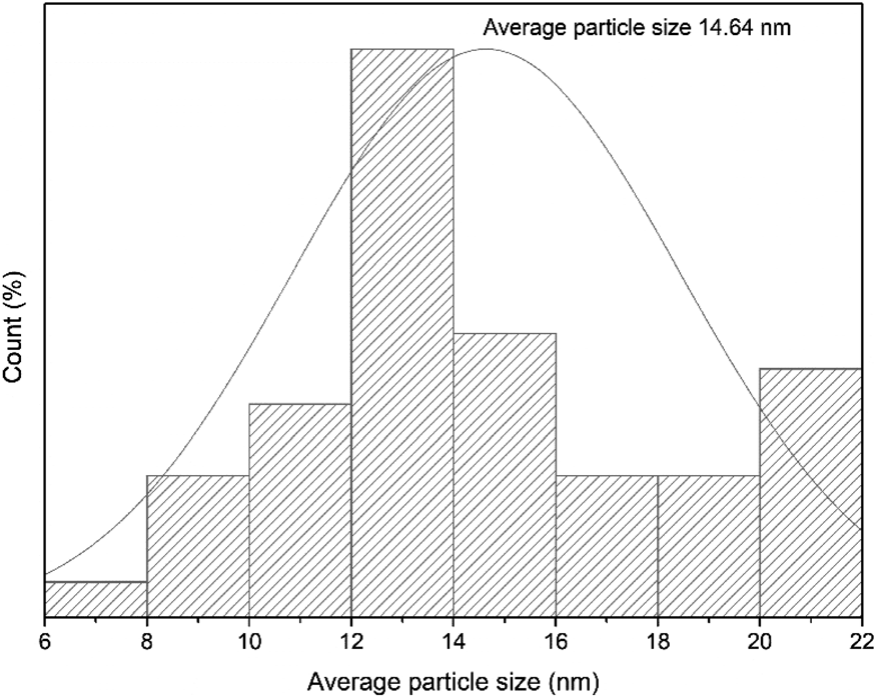
Histogram of MCNC@Au nanocomposite.

The morphologies of the nanoparticles within the MCNCs and the MCNC@Au were characterized using TEM. Fig. 3A and B show the typical morphology of the MCNCs and the MCNC@Au nanocomposite with Au typically showing higher contrast. The nanoparticle on the images looks aggregated which can be due to the magnetic properties it possesses. Due to the high electronic density of gold when compared to the magnetite, the shell structure of the particles is not visible on TEM. Therefore, to better differentiate between the Au and Fe_3_O_4_ nanoparticles, SEM and EDX were further employed for the characterization of MCNC@Au. Furthermore, Fig. 4 shows the histogram of MCNC@Au nanocomposite and the particle size ranged between 6 and 22 nm and the average particle size was 14.64 nm.

#### 3.1.3. SEM-EDX

**Fig. 5:**
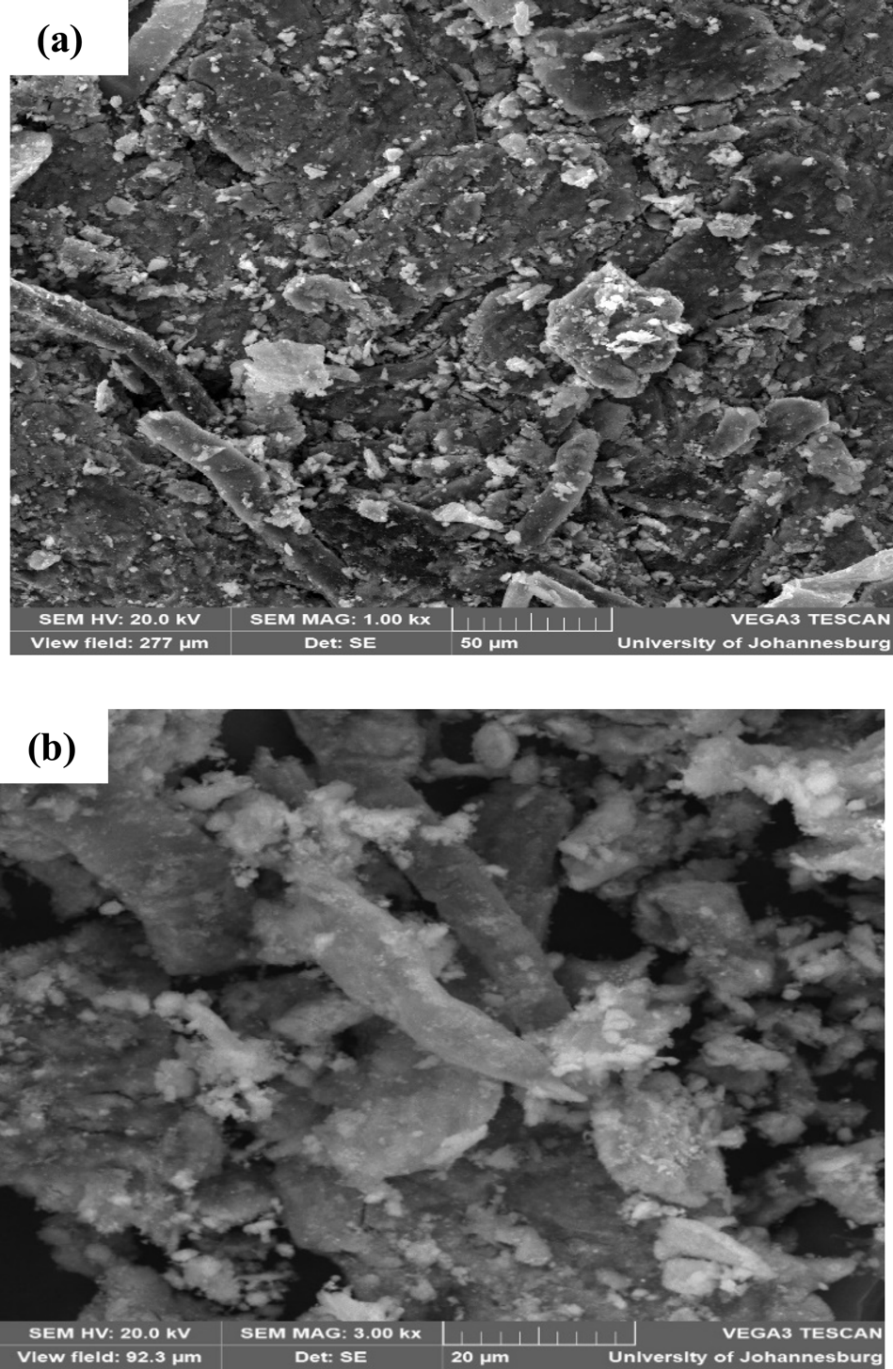
SEM image of (a) MCNC and (b) MCNC@Au.

**Fig. 6:**
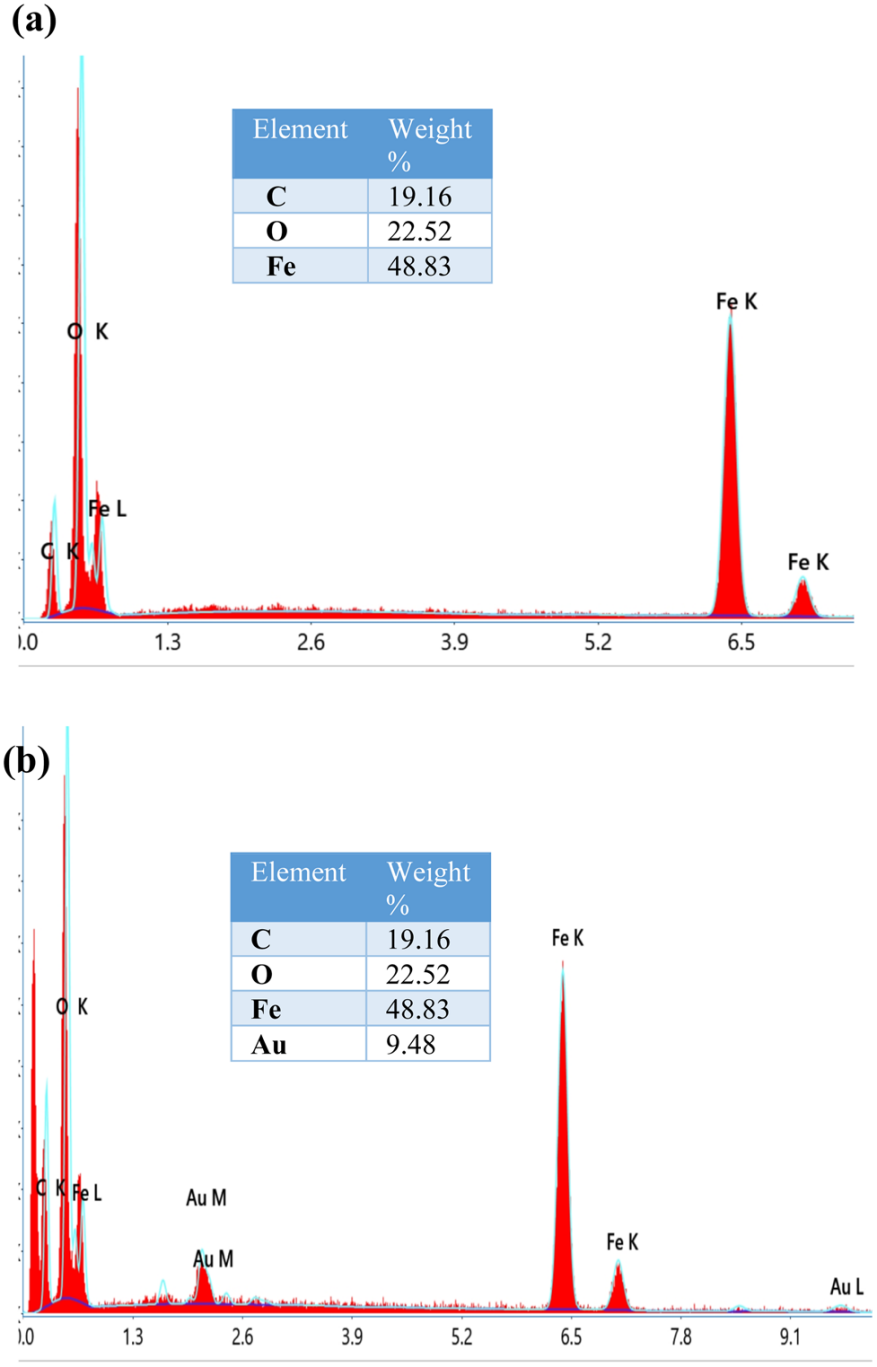
EDX spectrum and elemental composition of (a) MCNC and (b) MCNC@Au.

SEM and EDX were employed to further characterize MCNC and MCNC@Au nanocomposite. The presence of CNC was confirmed by the rod-shaped structure which appears in both SEM images (Fig. 5A and B) and the white spherical-shaped structure indicates the iron oxide attached to the CNC. To confirm the presence of gold in the synthesized nanocomposite, its EDX spectrum (Fig. 6A) was compared to that of MCNC (Fig. 6B). The presence of gold in the nanocomposite was confirmed with an elemental composition of 9.48 weight%.

#### 3.1.4. P-XRD

**Fig. 7:**
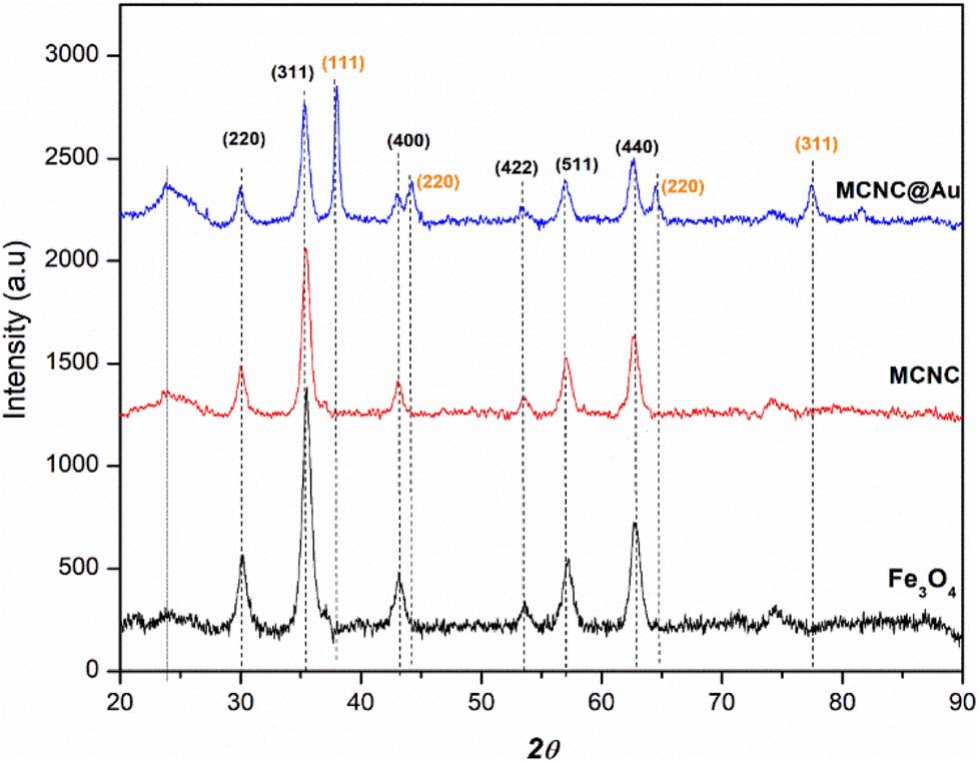
P-XRD patterns of Fe_3_O_4_, MCNC and MCNC@Au.

The XRD diffraction patterns of Fe_3_O_4_ nanoparticles (black) depict diffraction peaks at 30.10, 35.56, 43.03, 53.54, 57.18, and 62.83 2*θ* which can be indexed to the (220), (311), (400), (422), (511), and (440) planes of Fe_3_O_4_ in a cubic phase, respectively (Fig. 7). In the pattern of MCNC (red), two peaks at 16.77 and 23.6 2*θ* are attributed to (110) and (200) diffractions of cellulose, respectively. The diffraction peaks of MCNC@Au nanocomposite, indicated by the blue colour, are comparable to those of Fe_3_O_4_ nanoparticles, CNC, and Au nanoparticles reported in previous studies such as that conducted by Sedki and co-workers.^42^ These peaks were assigned at (111), (200), (220), (311), and (222) for Au (indicated by the orange Miller index), (220), (311), (511) and (440) for Fe_3_O_4_ and (110) and (200) for CNC. The penetration of X-rays through the thin gold-coated layer to the central Fe_3_O_4_ core revealed the diffraction peaks of Fe_3_O_4_.

### 3.2. Preliminary experiments

**Fig. 8:**
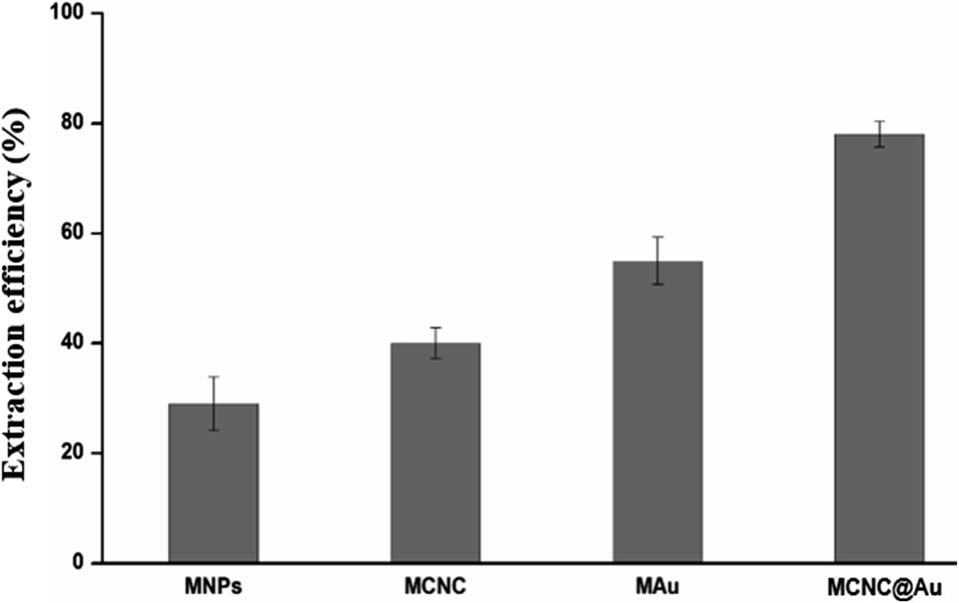
Effect of each magnetic nanomaterial present in the final nanocomposite were investigated on their performance for total sulfur extraction in fuel sample. Conditions: 20 mg sorbent, 20 mL of crude oil diluted to 20 ppm sulfur content with DCM, 35 min sorbent time at r.t., 200 μL MeOH as eluent solvent, 7 min elution time, dilution factor of 50 with water, *n* = 3.

To evaluate the effect of each magnetic nanomaterial present in the final nanocomposite, different magnetic nanomaterials such as magnetic nanoparticles (MNPs), magnetic cellulose nanocrystals (MCNC), magnetic gold nanoparticles (MAuNPs) and magnetic cellulose nanocrystals with gold (MCNC@Au) were evaluated for preconcentration of total sulfur. The obtained results (Fig. 8) showed that the presence of these magnetic nanomaterials in the nanocomposite produces a synergistic effect. For example, MAuNPs proved to have high extraction efficiency compared to MNPs and MCNC with an extraction efficiency of 55%. The highest extraction efficiency of MAuNPs was caused by the high chemical affinity of Au towards sulfur atoms.^28,29,33^ The MCNC with an extraction efficiency of 40%, showed that CNC doesn't only act as a support for iron oxide nanoparticles, but also plays a major role in enhancing the adsorption ability of the nanocomposite. On the other hand, MNPs with an extraction efficiency of 29%, showed that it doesn't only offer magnetic properties but also add a minor value to the adsorption ability of the nanocomposite. Lastly, MCNC@Au showed an extraction efficiency of 78%. This shows that the presence of all the components in the proposed nanocomposite is necessary with a specific role.

### 3.3. Multivariate optimization

#### 3.3.1. Screening of parameters

**Fig. 9:**
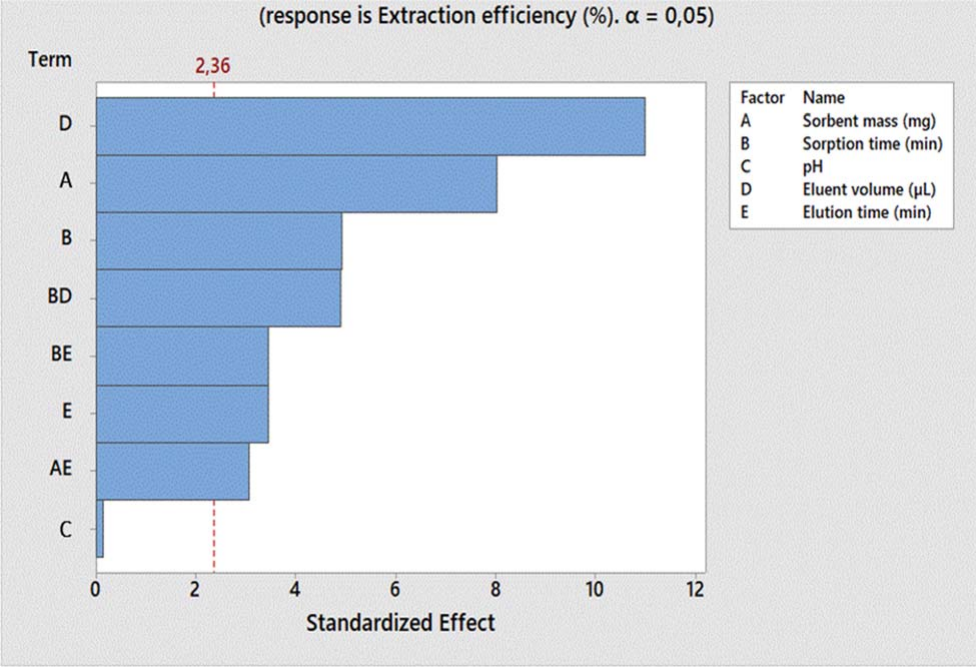
2-Level ½ fractional factorial design Pareto chart (*n* = 3) of total sulfur removal for the optimization of mass of sorbent, sorbent time, pH, eluent volume and elution time at 95% confidence level.

After the preliminary experiments, various parameters which could affect the adsorption reactions including sorbent mass (mg), sorption time (min), pH, eluent volume (μL) and elution time (min) were first screened using a 2-level ½ fractional factorial design. The analytical response of total sulfur was given in the form of extraction efficiency and the design matrix is displayed in Fig. 9. The design of experiment (DOE) results produced Pareto charts from ANOVA. The significant parameters are represented by the blue bars shown in Fig. 9 for each factor. The red line indicates the 95% confidence level. All the mentioned parameters were significant because they passed a 95% confidence level apart from pH.

#### 3.3.2. Response surface methodology

**Fig. 10:**
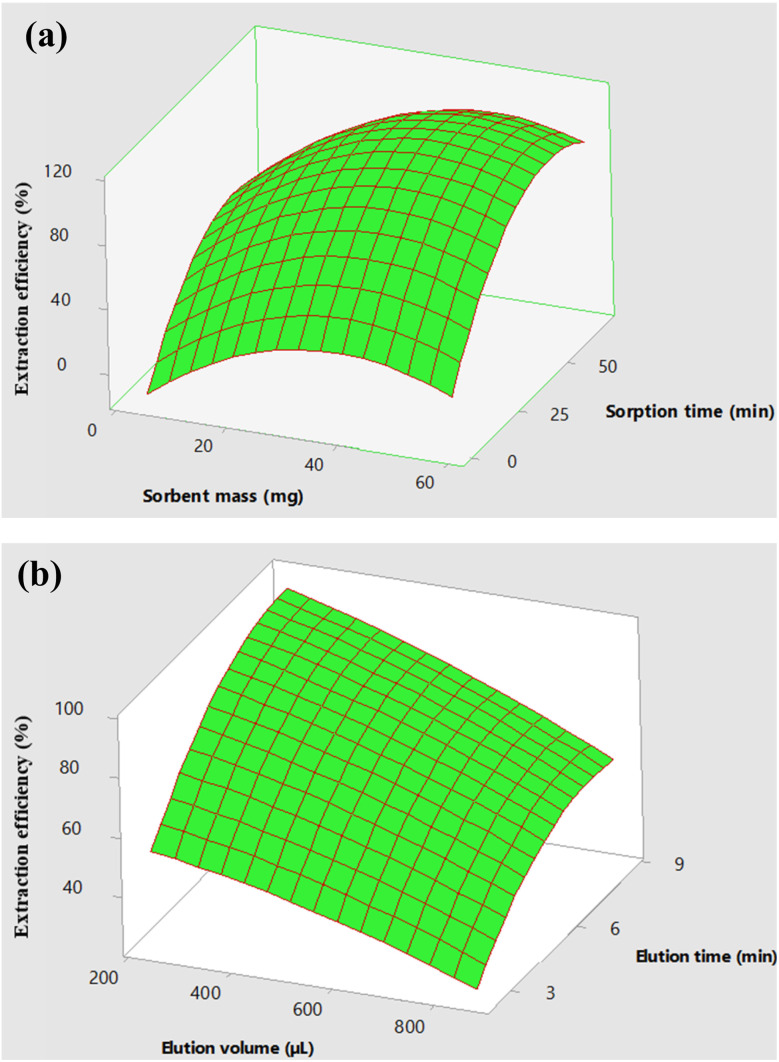
Response surface plot for (a) sorbent mass, sorption time and (b) elution volume, elution time *vs.* extraction efficiency of sulfur in liquid fuels.

The significant parameters of the adsorptive extraction experiments (sorbent mass and sorption time) and the desorption experiments (eluent volume and elution time) were further optimized using the central composite design (CCD). The CCD was chosen based on being mostly reported in literature and it is the most reliable. For extraction experiments, the parameter (pH) that was statistically insignificant, was kept at 6.5 throughout the experiments. The response surface plots (Fig. 10A and B) were used to evaluate the effects of sorbent mass, sorbent time, eluent volume and elution time on the analytical response. Based on the quadratic equations and the surface plot (Fig. 10A), the optimum conditions for extraction experiments were chosen to be 27.5 mg sorbent mass and 35 minutes sorption time with 200 μL eluent volume and 7 min elution time kept constant. On the other hand, based on the quadratic equations and the surface plot (Fig. 10B), the optimum conditions for desorption experiments were chosen to be 200 μL eluent volume and 8 min elution time with sorption mass and sorption time kept constant at 27.5 mg and 35 minutes, respectively.

### 3.4. Analytical figures of merits

In order to determine the efficiency of the proposed m-SPE-ICPOES method compared to the previously reported studies, the analytical figures of merit of the proposed m-SPE method were investigated under the established optimum conditions. The analytical features investigated include sensitivity, correlation coefficient (*R*^2^), limit of detection (LOD), limit of quantification (LOQ), method detection limit (MDL), method quantification limit (MQL), accuracy and precision. This study was achieved by preparing and extracting six increasing calibration standard concentrations (5, 10, 15, 20, 25 and 30 ppm) of sulfur in DCM in triplicates and then plotted against intensity to produce a linear graph. The graph provided linearity information such as *R*^2^ and method gradient (which is equivalent to the sensitivity of the analyte). The sensitivity was found to be 5695.7 cps L mg^−1^ and *R*^2^ was 0.9991 as presented in Table 1. The standard deviation was obtained from 20 blank samples (methanol), and it was used, together with a calibration gradient, to calculate the LOD and LOQ (see eqn (2) and (3)). LOD is referred to as the lowest concentration at which detection is feasible and at which a sample can be consistently distinguished from a blank. Conversely, LOQ is the lowest quantity of analyte in a sample that can be accurately and precisely quantified. The calculated LOD and LOQ were then used to calculate the method detection limit (MDL) and method quantification limit (MQL) (see eqn (4) and (5)). The results for all the investigated analytical figures of merit are presented in Table 1. Furthermore, the analytical figures of merit of the newly developed m-SPE method were then compared with those of various reported studies on sample preparation methods of fuel oil matrices prior to the ICP OES-based method. The data in Table 2 shows that this newly developed m-SPE method can be the most appropriate sulfur-extracting method in the future based on its accuracy, precision, sensitivity, LOD and LOQ.^43–45^2
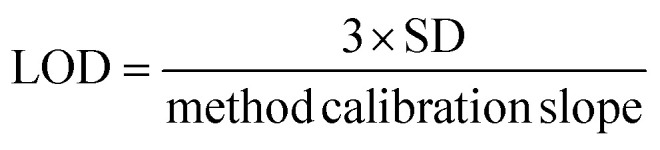
3
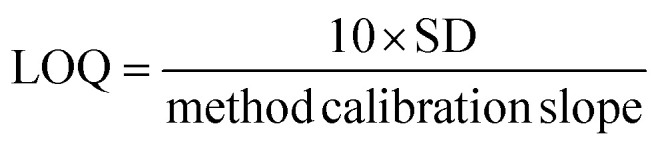
4
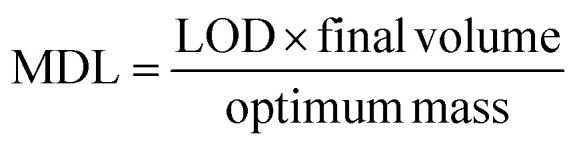
5
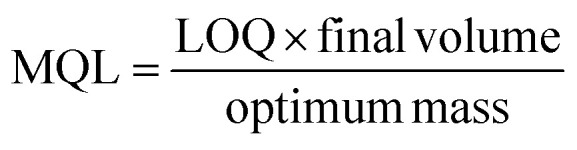


**Table 1:** Analytical features of merit of the proposed MSPE-ICP-OES method for the total determination of total sulfur under the established optimum conditions

Analytical features	Specification
Sensitivity (cps L mg^−1^)	5695.7
Correlation coefficient (*R*^2^)	0.9991
LOD (mg L^−1^)	0.039
LOQ (mg L^−1^)	0.129
MDL (μg g^−1^)	0.014
MQL (μg g^−1^)	0.047
Accuracy (%)	98.8
Precision (%)	1.7

**Table 2:** Comparison of analytical figures of merit of the proposed m-SPE method with literature reported studies

Matrix	Sample preparation method	Accuracy (%)	Precision (%)	LOD (mg L^−1^)	LOQ (mg L^−1^)	Detection technique	Ref.
Crude oil, diesel, gasoline and kerosene	m-SPE	98.8	1.7	0.039	0.129	ICP-OES	This work
Biodiesel	Dilution	95	5<	0.40	1.3	ICP-OES	43
Diesel, gasoline and kerosene	Emulsion preparation using nitric acid, Triton X-100 and water	97–103	4.3<	0.72	2.4	ICP-OES	44
Diesel, gasoline and kerosene	m-SPE	94–101	N/A	0.15	0.5	ICP-OES	20
Crude oil distillation residues	Microwave-induced combustion	92–101	8.3<	2.0	6.67	ICP-OES	45

### 3.5. Reusability studies

**Fig. 11:**
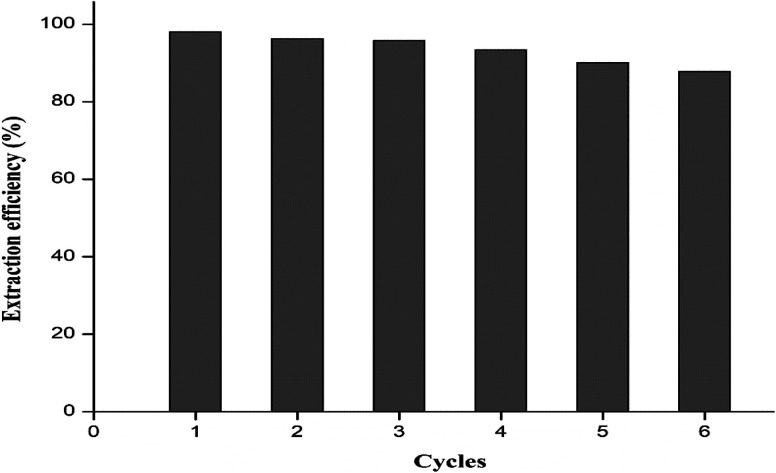
The reusability studies for MCNC@Au nanocomposite on sulfur extraction in fuel oil samples.

The reusability of the MCNC@Au nanocomposite was examined for adsorptive extraction of total sulfur and seven cycles were conducted under the optimum conditions. After every cycle, the nanocomposite was magnetically removed from the matrix, washed, and then dried at 70 °C for 1 hour. From the results presented in Fig. 11, an extraction efficiency of 87.8% was obtained after six cycles indicating that MCNC@Au nanocomposite as an adsorbent may be used as a reusable adsorbent for the effective extraction of sulfur.

### 3.6. Anti-interference ability of the method

Other chemical contaminants (such as PAHs), organomercury compounds, and metallic mercury were present in the crude oil CRM used in this study. Nonetheless, in the presence of the previously indicated matrix chemicals, the proposed method had an extraction efficiency of 98% and was quite selective for sulfur.

### 3.7. Application of the m-SPE method on commercial fuel oil samples

The optimum and validated m-SPE sample preparation method was applied to various selected commercial fuel oil samples (crude oil, diesel, gasoline and kerosene) to determine the concentration levels of total sulfur. Two different samples (A and B) were analyzed for each fuel oil. The extraction of total sulfur was conducted under the established optimum conditions prior to the ICP-OES analysis and the results are reported in Table 3 below, revealing a total sulfur content range between 7.32 and 15.7 μg g^−1^. Both crude oil samples proved to have high total sulfur concentration levels (15.7 and 13.2 μg g^−1^) compared to the other fuel oil samples, diesel (9.12 and 7.32 μg g^−1^), gasoline (8.41 and 9.15 μg g^−1^) and kerosene (9.7 and 9.1 μg g^−1^). Ultimately, the obtained total sulfur concentration levels are within the accepted standards except for those of crude oil. Therefore, there is an urgent need to reduce the sulfur concentration levels in particular crude oil prior to commercialization by applying effective desulfurization methods or making sure that the storage and transportation tanks (which may contribute to the high sulfur concentration levels through contamination) are well taken care of to minimize the chances of having contaminated commercial crude oil. Furthermore, our synthesized nanocomposite can be used as an adsorbent to further reduce the sulfur content in fuel oils to ultra-low sulfur concentration through adsorptive desulfurization.

**Table 3:** Concentration levels of total sulfur (in ppm) in real fuel oil samples after m-SPE-ICP-OES analysis

Commercial fuel oils	Sample A (μg g^−1^)	Sample B (μg g^−1^)
Crude oil	15.7 ± 0.02	13.2 ± 0.05
Diesel	9.12 ± 0.03	7.32 ± 0.01
Gasoline	8.41 ± 0.02	9.15 ± 0.06
Kerosene	9.7 ± 0.04	9.1 ± 0.02

## 4. Conclusions

The new magnetic cellulose gold nanocomposite was successfully synthesized and characterized using FT-IR, XRD, SEM-EDX and TEM. This nanocomposite was successfully used in the development of the simple and rapid m-SPE method for the determination of total sulfur concentrations in liquid fuel matrices prior to the ICP-OES analysis. The developed m-SPE method was quite precise and had a good accuracy of above 98% and with good sensitivity as well. The method also reported very low LOD (0.039 mg L^−1^) and LOQ (0.129 mg L^−1^) values. Thus, by enhancing sensitivity and preventing the degradation of hot plasma characteristics and extinction issues, this proposed method significantly improves ICP-OES experiments. Furthermore, with the obtained high concentrations of total sulfur in the analysed commercial crude oil samples, the newly synthesized nanocomposite could also be used as an effective adsorbent in adsorptive desulfurization methods.

## Data availability

The data supporting this article have been included as part of the ESI.†

## Conflicts of interest

There are no conflicts to declare.

## Supplementary Material

RA-014-D4RA04427D-s001
