# Supplementary material for: Selective and precise solid phase extraction based on a magnetic cellulose gold nanocomposite followed by ICP-OES analysis for monitoring of total sulfur content in liquid fuels
Source: RSC Adv. 2024 Sep 2;14(38):27990–8. doi: 10.1039/d4ra04427d (PMC11367618; doi:10.1039/d4ra04427d)
Supplement: RA-014-D4RA04427D-s001 [file RA-014-D4RA04427D-s001.pdf]

Supplementary Information (SI) for RSC Advances.
This journal is © The Royal Society of Chemistry 2024

Selective and precise solid phase extraction based on magnetic cellulose gold nanocomposite followed by ICP-OES analysis for monitoring of total sulfur content in liquid fuels

Kgomotso G. Mabena[a], Philiswa N. Nomngongo[b], Nomvano Mketo[a1]

[a]*Department of Chemistry, College of Science and Engineering and Technology, Florida Science Campus, University of South Africa, Roodepoort, 1710, Johannesburg, South Africa*

[b]*Department of Chemical Sciences, University of Johannesburg, PO Box 17011, Doornfontein 2028, Johannesburg, South Africa.*

Table S1. Operating parameters and conditions of ICP-OES for total sulfur analysis

**Parameters**	**Conditions**
RF Power (W)	1200 W
Auxiliary gas flow rate(L/min)	1.5 L/min
Plasma gas (Ar) flow rate (L/min)	15.0 L/min
Pump speed (rpm)	85 rpm
Peri-pump speed analysis (s)	15 s
Stabilization time (s)	15 s
Nebulizer gas flow rate (L/min)	0.75L/min
Elemental wavelength	S (182.562)
Replicates	3

[1] Corresponding author: Email address: nomvano.mketo@gmail.com or mketon@unisa.ac.za; Tel: +27114712032

Table S2. The minimum and maximum amount used for different parameters for screening.

Variable	Minimum (-)	Maximum (+)
Mass of sorbent (mg)	5	50
Sorption time (min)	10	60
pH	4	9
Eluent volume (μL)	100	1000
Elution time (min)	4	10
